# Prevalence, patterns and correlates of alcohol consumption and its’ association with tobacco smoking among Sri Lankan adults: a cross-sectional study

**DOI:** 10.1186/1471-2458-14-612

**Published:** 2014-06-17

**Authors:** Prasad Katulanda, Chathuranga Ranasinghe, Amila Rathnapala, Nalika Karunaratne, Rezvi Sheriff, David Matthews

**Affiliations:** 1 Faculty of Medicine, University of Colombo, PO box 25, Kynsey road, Colombo 10, Sri Lanka; 2 Oxford Centre for Diabetes Endocrinology & Metabolism, Churchill Hospital, Old road, Headington, Oxford OX3 7LJ, UK

**Keywords:** Alcohol, Tobacco, Smoking, Prevalence, Sri Lanka

## Abstract

**Background:**

Most studies on alcohol consumption carried out in Sri Lanka are limited to single/few provinces in the island. The objective of this study was to determine the prevalence, patterns and correlates of alcohol consumption among a larger sample of adults in Sri Lanka.

**Methods:**

This cross-sectional study was conducted in seven of all nine provinces in Sri Lanka, between 2005 and 2006. A nationally representative sample of 5000 adults aged ≥18 years was selected using multi-stage random cluster sampling. Data of 4532 participants were collected using an interviewer administered questionnaire. Data analysis included chi-squared test, multiple logistic regression analysis and Spearman correlation using Stata/SE 10.0 (StataCorp LP., Texas, USA) software package.

**Results:**

Males were 40%; mean age was 46.1 years (±15.1). The overall, urban and rural prevalence (95% CI) of current drinking was 23.7% (21.7 – 25.7), 29.5% (25.7 – 33.3) and 22.2% (19.8 – 24.7) respectively (p = 0.001). Current (M: 48.1%, F: 1.2%, p < 0.0001) and former (M: 21.4%, F: 0.7%, p < 0.0001) drinking was much higher in males. The highest prevalence of drinking in males (58.9%) and females (2.2%) was in the 30 – 39 and <20 year age groups respectively. Lowest prevalence in men (24.6%) and women (0%) was in the >70 years age-group. Hazardous drinking was seen in 5.2% of men and 0.02% of women. Male sex, urban living and current smoking correlated with both current and hazardous drinking. Lower level of education, and age >70 years positively correlated with hazardous drinking.

**Conclusions:**

Alcohol is predominantly a problem in Sri Lankan males. In males, both current and hazardous drinking positively correlated with urban living, white collar occupation, Burgher ethnicity and current smoking. Hazardous drinking positively correlated with lower level of education and older age. The data shown here are useful in planning interventions simultaneously targeting alcohol and tobacco.

## Background

According to the World Health Organization (WHO) estimations; two billion people worldwide consume alcohol and 76.3 million are afflicted with diagnosable alcohol use disorders [1]. The WHO has attributed 3.7% of all deaths in all age groups to alcohol [2] (6.1% in men and 1.1% in women). Also excessive use of alcohol can lead to physical, psychological and social harm [2-7].

Sri Lanka is a middle income country with a population of 20 million people [8]. Historically the Sri Lankan villagers had consumed ‘;toddy’, a fermented beverage with low alcohol content for many centuries [9]. Toddy is produced using the sugary juice obtained by tapping the sap of coconut (*Cocos nucifera*), palmyrah (*Borassus flabellifer*) or ‘;Kitul’ (*Caryota urens*). The use of alcoholic spirits became popular after Sri Lanka was colonized by the Europeans: starting with the Portuguese in 1505, followed by the Dutch and the English. This trend continued since gaining independence in 1948 [5,9]. The main licit alcoholic spirit is ‘;Arrack’, a distilled beverage produced from coconut or sugar cane. Many low income groups consume illicit spirits which are called ‘;Kasippu’ or ‘;Moonshine’. Recently, beer drinking has been gaining popularity especially among younger age groups [5].

According to the WHO Gender, Alcohol and Culture: An International Study (GENACIS) in 2002 (n = 1201), 53.1% of the men and 6.4% of women in Sri Lanka were current drinkers (those who had consumed alcoholic beverages during the past 12 months) [9]. Few other studies conducted in Sri Lanka have been of small sample sizes, confined to a limited geographical region or not published internationally [10]. According to these studies, the prevalence of alcohol use ranged between 37.7 – 52.5% in men and 1.6 – 5.0% in women [10]. In a study among teenagers, the prevalence of alcohol was reported as 21.2% in men and 3.3% in women [11]. A hospital based study in 1999 showed that 24% of male deaths were alcohol related [12]. Also the alcohol use has been shown to be a risk factor for suicide, accidental poisoning, snake bite and domestic violence in Sri Lanka [13-16].

Sri Lanka is undergoing a rapid socio-demographic transition [17,18]. A study conducted in two urban and rural districts in the country revealed that the prevalence of current drinking in the urban areas (32.9%) was significantly higher than in rural areas (20.8%) [19]. In literature, there is a paucity of large studies on the epidemiology of alcohol consumption in Sri Lanka, which is a drawback in planning and implementing strategies on alcohol related problems. We aimed to determine the prevalence, patterns and socio-economic correlates of current and hazardous alcohol use and their association with tobacco smoking among a larger sample of adults in Sri Lanka.

## Methods

The Sri Lanka Diabetes and Cardiovascular Study (SLDCS) was conducted by the Diabetes Research Unit of the University of Colombo with the Oxford Centre for Diabetes Endocrinology and Metabolism UK, and was approved by the Ethical Review committee of the Faculty of Medicine - University of Colombo, Sri Lanka. All participants provided informed written consent. Data collection was between August 2005 and September 2006.

### Study population and sampling

SLDCS was conducted, in seven of all nine provinces in Sri Lanka and included non-institutionalized adults aged 18 years and above (≥18 years). The sample was selected using a cross sectional multistage random cluster sampling method. The total sample frame was approximately 14 million adults, ≥18 years of age living in 12 018 ‘;Village Officer’ units (the smallest government administrative unit). A final sample size of 5000 was calculated after adjusting for an estimated non-response rate of 25%. Probability-proportional to-size (PPS) technique was used to determine the sample sizes for individual provinces and rural and urban sectors in each province. The total sample was recruited from 100 clusters, where clusters were selected by a computer-generated random number list from the ‘;Village Office Units’. Fifty households from each cluster were selected to ensure a wider representation of the population. Selected households were visited by the study team and simple random selection of participants was done after informed verbal consent. An eligible adult, aged ≥18 years was selected from all eligible adults of each consenting household. Those who were pregnant, acutely ill or who declined participation were excluded. Detailed method of sampling and data collection has been reported previously [20].

### Data collection, terms and classifications

Temporary data collection centers were established within each cluster. The selected subjects were invited to visit the centers at a predetermined date and time. Privacy and confidentiality was strictly maintained during data collection. An interviewer administered questionnaire (Additional file 1) was used to collect data, administered by a trained group of medical graduates. The questionnaire was pre-tested to evaluate the face-validity and to ensure whether the subjects understood what the investigators intended to know. Information regarding socio-demographic factors, smoking status and alcohol intake was recorded. The participants were asked whether they have consumed at least ‘;one drink’ of any form of alcoholic beverage (‘;Arrack’, ‘;Kassippu’, Whisky, Gin, Brandy, beer, toddy, wine or other alcoholic beverages) at least once during their lifetime. If the answer was ‘;no’ they were considered as lifetime abstainers. If the answer was ‘;yes’, they were asked whether they had abstained from consuming alcohol for the last 6 months (prior to being selected to the present study). Those answered ‘;yes’ were considered as ‘;former drinkers’ and the others as ‘;current drinkers’. The quantity of alcohol used was recorded per week, using an annexure to the main questionnaire (Additional file 2) and a reference table (Additional file 3) carried by each interviewer. The amount of alcohol was recorded in units per week (1 unit = 8 g of alcohol) based on the average alcohol content of different alcoholic beverages; one drink (25–30 ml) of arrack/whisky, 50 ml of illicit alcohol [21], half a pint of beer/toddy and a small glass of wine (175 ml) were considered to be equivalent to a unit of alcohol (Additional file 3). Consuming ≥ 21 units of alcohol by a male and ≥ 14 units by a female during a week was considered as ‘;hazardous drinking’ [22,23].

Participants were considered to be ‘;current smokers’, if they had been smoking any form of tobacco (cigarettes, bidi or cigars) either every day or on some days at the time of the study, within the preceding 6 months. Those who had successfully quit at least 6 months before the data collection were considered ‘;ex-smokers’ and those who have not smoked tobacco were considered ‘;never smokers’ [24].

Sinhalese are the predominant ethnic group in Sri Lanka, followed by the Sri Lankan Tamils (Descendants from South India over many centuries) and Sri Lankan Moor (descendants of Arab traders who are followers of Islam). Plantation Tamil (People arrived from South India during the last century for Tea and Rubber plantation) and Burgher (Descendants of Dutch settlers) are other minority groups.

Income levels were categorized based on monthly family income in Sri Lankan rupees (LKR). International Standard Classification of Occupation (ISCO 88) was used to categorize the occupation of the participants. Educational level was divided into three categories based on number of years of full time education (school or higher education). Urban and rural sectors were selected according to the categorization by the Sri Lankan Government.

### Statistical analysis

All data were double-entered and cross checked for consistency. Data were analyzed using the survey commands in Stata/SE 10.0 (StataCorp LP., Texas, USA) software package taking into account the stratified cluster sampling [25]. Sampling weights were assigned to account for the differences in response at cluster and household levels as well as for gender specific response. Data on alcohol consumption are presented for age categories, gender, sector of residence, educational status, occupation and income level. Data are presented as percentages and 95% CI whenever appropriate. Chi-squared test was used to compare the prevalence between different socio-demographic categories. Multiple logistic regression analysis was performed to examine the association of underlying factors for current, hazardous, former and never drinking. Spearman correlation was used to determine associations between smoking and alcohol use. P values <0.05 were considered significant.

## Results

Among the 5000 selected, 4532 participated in the study with a response rate of 91%. This report is based on 4491 participants with complete data. The mean age of the sample was 46.13 ± 15.1 years. Males were 40% (n = 1788) of the total sample and 17.7% of them were from the urban sector. In the study sample 85.4% were Sinhalese, 3.6% Sri Lankan Tamil, 3.8% Plantation Tamil, 6.9% Sri Lankan Moor and 0.3% were others. According to the 2001 census in Sri Lanka, urban population was 15% and the proportionate population sizes of Sinhalese (82%), Sri Lankan Tamils (4.3%), Plantation Tamils (5.1%), Sri Lankan Moor (7.9%) and others (0.7%) closely resembled the distribution in the sample [8].

### Overall and ethnic specific alcohol use

Overall, 65.7% (95% CI, 63.5 – 67.8%) were lifetime abstainers (Male: 30.4%, 95% CI 26.8 – 34.0%; Female: 98.1%, 95% CI 97.3 – 98.9%, p < 0.0001). The total prevalence of current and former alcohol use was 23.7% (95% CI, 21.7 – 25.7%) and 10.6% (95% CI 9.4 – 11.9%) respectively (Table 1). The prevalence of current drinking in males was much higher than females (M: 48.1%, 95% CI, 44.3 – 52.0%; F: 1.2%, 95% CI 0.6 – 1.8%, p < 0.0001). Among the different ethnic groups, in males; Sri Lankan Tamils had the highest prevalence of current drinking (67.1%, 95% CI 52.6 – 81.6%) and the Sri Lankan Moors had the lowest (7.3%, 95% CI 1.3 – 13.4%) (Table 2). In males ethnic specific differences in current (p <0.0001) and former dinking (p <0.05) were significant; whereas in females it was not. The highest prevalence of female drinking was seen among the Burgher women (30.8%) whilst none of the Sri Lankan Moor women had self-reported drinking.

**Table 1:** Alcohol consumption in urban and rural adults (Sri Lanka, 2005 – 2006)

**Sector**	**Category**	**number**	**Current drinking %, (95% CI)**	**Former drinking %, (95% CI)**
**Urban**	**Male**	312	57.6 (51.2-63.9)	16.0 (10.9-21.2)
	**Female**	487	2.9 (1.1-4.8)	1.6 (0.5-2.8)
	**All**	799	29.5 (25.7-33.3)	8.6 (5.9-11.3)
**Rural**	**Male**	1466	45.7 (41.1-50.3)	22.9 (20.1-25.6)
	**Female**	2226	0.7 (0.1-1.4)	0.4 (0.1-0.8)
	**All**	3692	22.2 (19.8-24.7)	11.2 (9.8-12.6)
**Overall**	**Male**	1778	48.1 (44.3-51.9)	21.4 (19.1-23.8)
	**Female**	2713	1.2 (0.6-1.8)	0.7 (0.3-1.0)
	**All**	4491	23.7 (21.7-25.7)	10.6 (9.4-11.9)

Data are % (95% CI), Confidence interval (CI).

**Table 2:** Alcohol consumption among adults by the age, sex and ethnicity (Sri Lanka 2005 – 2006)

		**Male**			**Female**		
		**Total (n)**	**Current (%)**	**Former (%)**	**Total (n)**	**Current (%)**	**Former (%)**
**Age group (years)**	<20	53	34.6 (18.2-51.0)	14.2 (4.8-23.5)	45	2.2 (0.2-6.7)	0.0 (0.0-0.0)
	20 – 29	260	48.0 (40.9-55.0)	18.9 (13.9-23.8)	384	1.3 (0.4-2.9)	0.8 (0.1-1.7)
	30 – 39	335	58.9 (52.6-65.3)	17.1 (12.8-21.4)	553	1.8 (0.5-3.1)	0.6 (0.1-1.2)
	40 – 49	418	50.3 (44.0-56.6)	18.5 (14.0-22.9)	674	2.0 (0.5-3.6)	0.4 (0.1-0.8)
	50 – 59	350	52.4 (45.7-59.0)	15.9 (12.0-19.8)	547	0.2 (0.1-0.6)	0.7 (0.2-1.6)
	60 – 69	212	38.2 (30.9-45.6)	35.6 (28.3-42.8)	326	0.7 (0.6-2.0)	1.3 (0.2-2.4)
	≥ 70	150	24.6 (15.5-33.6)	42.5 (32.7-52.2)	184	0.0 (0.0-0.0)	0.5 (0.5-1.4)
	All	1778	*p <0.0001	*p <0.0001	2713	*p < 0.005	*p < NS
**Ethnicity**	Sinhalese	1524	49.4 (46.2-52.7)	22.6 (20.1-25.1)	2356	1.1 (0.6-1.7)	0.7 (0.3-1.0)
	Sri Lankan Tamil	59	67.1 (52.6-81.6)	8.7 (2.4-15.0)	77	1.8 (0.1-5.4)	0.0 (0.0-0.0)
	Plantation Tamil	76	65.6 (53.3-77.9)	13.4 (6.3-20.4)	88	2.5 (0.1-6.7)	2.2 (0.1-4.4)
	Burgher	3	64.0 (0.1-129.9)	0.0 (0.0-0.0)	8	30.8 (5.5-56.1)	11.2 (0.4-22.0)
	Sri Lankan Moor	116	7.3 (1.3-13.4)	20.1 (11.0-29.1)	184	0.0 (0.0-0.0)	0.0 (0.0-0.0)
	All	1778	^+^p < 0.0001	^+^p < 0.05	2713	^+^p = NS	^+^p = NS

*p values for age specific differences in current drinking and former drinking in males and females**.**

+p values for ethnic specific differences in current drinking and former drinking in males and females.

Data are % (95% CI), Current-current drinkers, Former-past drinkers, Confidence interval (CI), Not Significant (NS), Number of participants (n).

### Age and sector specific alcohol consumption

In males, the highest prevalence of current drinking (58.9%) was in the 30 – 39 year age group while those more than 70 years had the lowest, with the prevalence declining after 60 years of age (Table 2). In women, the highest prevalence of current drinking was in the youngest age group (2.2%) and the lowest (zero prevalence) was in the oldest. In both males and females age specific differences in current dinking was significant (Male p <0.0001, Female p < 0.005), whereas age specific differences in former drinking was significant only among males (p <0.0001). In men, highest prevalence of former drinking was seen in >70 years age group, whereas in women it was 60–69 years. Overall, the urban sector had higher prevalence of current drinking compared to rural (urban 29.5%, 95% CI 25.7 – 33.3%, rural 22.2%, 95% CI 19.8 – 24.7%, p < 0.0013) (Table 1). The higher current drinking in the urban population was similarly present in both males (urban 57.6% vs. rural 45.7%, p < 0.0001) and females (urban 2.9% vs. rural 0.7%, p < 0.0001). In contrast, the overall (p = 0.1310) and the male (p = 0.041) prevalence of former alcohol use was higher in the rural sector compared to urban.

### Severity and hazardous drinking

Among the current drinkers in this study, 80% consumed less than 7 units of alcohol per week (18.8% of the total sample). Only one woman reported consuming more than 7 units per week in the whole sample. Among drinking men, 79% consumed less than 7 units per week. However, the second highest prevalence was seen in the >21 units category. Both overall and males drinking at intermediate levels were less than these two categories. In this sample, the prevalence of overall, male and female hazardous drinking were 2.5% (95% CI, 1.9 – 3.1%), 5.2% (95% CI 3.9 – 6.4%) and 0.02% (95% CI 0.018 – 0.055%) respectively (Table 3). There were no differences in the prevalence of hazardous drinking in urban and rural sectors (Urban: 2.7% versus Rural: 2.4%, p = 0.718).

**Table 3:** Amount of alcohol consumption among adults (Sri Lanka, 2005 – 2006)

**Sector**	**Category**	**Number**	**Number of units of alcohol per day**			
			**< 7**	**7 – 13**	**14 - 20**	**≥21**
**Urban**	**Male**	312	46.2 (40.6-51.8)	4.1 (1.6-6.5)	1.9 (0.5-3.2)	5.4 (2.7-8.2)
	**Female**	487	2.8 (1.1-4.6)	0.0 (0.0-0.0)	0.0 (0.0-0.0)	0.1 (0.01-0.3)
	**All**	799	23.9 (20.5-27.3)	2.0 (0.8-3.2)	0.9 (0.2-1.6)	2.7 (1.3-4.1)
**Rural**	**Male**	1466	35.8 (32.0-39.5)	2.3 (1.4-3.2)	2.6 (1.6-3.6)	5.1 (3.6-6.5)
	**Female**	2226	0.7 (0.1-1.4)	0.0 (0.0-0.0)	0.0 (0.0-0.0)	0.0 (0.0-0.0)
	**All**	3692	17.5 (15.5-19.5)	1.1 (0.7-1.5)	1.2 (0.7-1.7)	2.4 (1.8-3.1)
**Overall**	**Male**	1778	37.9 (34.8-41.0)	2.6 (1.8-3.5)	2.4 (1.6-3.3)	5.2 (3.9-6.4)
	**Female**	2713	1.2 (0.6-1.8)	0.0 (0.0-0.0)	0.0 (0.0-0.0)	0.02 (0.01-0.05)
	**All**	4491	18.8 (17.1-20.5)	1.3 (0.9-1.7)	1.2 (0.8-1.6)	2.5(1.9-3.1)

Data are % (95% CI), Confidence interval (CI).

### Associations of current and hazardous drinking with the level of education, income and occupation

In men, the highest levels of current drinking were seen in the group with middle level of education; (50.7%) (Table 4). In contrast, the highest prevalence of current drinking in women (2.6%) was seen in the group with the highest education. When comparing occupational categories in both men and women, the highest prevalence of current drinking was seen among ‘;senior officials and managers’. In men, the lowest prevalence was in the ‘;unemployed’ (36.1%) and in women zero prevalence was seen in those from ‘;armed forces’, the ‘;plant and mechanic operators/assemblers’ and ‘;skilled agricultural and fisheries workers’. In both sexes, the highest income group had the highest prevalence of current drinking.

**Table 4:** Prevalence of current and hazardous alcohol consumption by the level of education, occupation and income

	**Educational and occupational category**	**Male**			**Female**		
		**N**	**Current (%)**	**Hazardous (%)**	**N**	**Current (%)**	**Hazardous (%)**
**Education**	≤ 5 years	363	43.4 (36.1-50.7)	8.5 (4.9-12.1)	709	0.7 (0.1-1.4)	0.0 (0.0-0.0)
	6 – 11 years	1094	50.7 (46.1-55.3)	5.3 (3.8-6.8)	1575	1.1 (0.4-1.8)	0.0 (0.0-0.0)
	≥ 12 years	320	45.0 (37.7-52.4)	1.1 (0.0-2.2)	427	2.6 (1.0-4.2)	0.0 (0.0-0.0)
**Occupation**	Senior officials and managers	10	79.8 (59.5-100.0)	11.1 (0.3-32.0)	7	41.3 (12.7-69.9)	0.0 (0.0-0.0)
	Professionals	51	41.1 (23.3-59.0)	0.0 (0.0-0.0)	57	2.5 (0.2-6.2)	0.0 (0.0-0.0)
	Technical and associated professionals	76	69.0 (57.0-81.0)	4.5 (0.2-9.1)	18	4.8 (0.3-14.3)	0.0 (0.0-0.0)
	Clerks	28	54.8 (33.4-76.3)	0.0 (0.0-0.0)	42	1.6 (-1.5-4.6)	0.0 (0.0-0.0)
	Sale and service workers	223	52.0 (42.0-62.0)	3.9 (1.3-6.4)	78	4.3 (-1.0-9.6)	0.0 (0.0-0.0)
	Skilled agricultural and fishery workers	225	37.2 (27.9-46.5)	4.7 (2.3-7.1)	86	0.0 (0.0-0.0)	0.0 (0.0-0.0)
	Craft and related workers	59	41.1 (27.9-54.4)	6.3 (0.2-12.3)	48	5.6 (1.0-13.7)	0.0 (0.0-0.0)
	Plant and mechanic operators and assemblers	100	64.6 (53.9-75.4)	6.1 (1.9-10.4)	13	0.0 (0.0-0.0)	0.0 (0.0-0.0)
	Elementary occupations	480	56.8 (50.3-63.4)	7.3 (4.2-10.5)	364	1.6 (0.3-3.4)	0.1 (0.01-0.4)
	Armed Forces	11	56.7 (24.8-88.5)	0.0 (0.0-0.0)	3	0.0 (0.0-0.0)	0.0 (0.0-0.0)
	No regular employment or unemployed	514	36.1 (31.0-41.3)	4.5 (2.3-6.3)	1997	0.7 (0.3-1.1)	0.0 (0.0-0.0)
**Income category (LKR 000)**	< 7.0	866	43.2 (37.3-49.1)	5.6 (3.6-7.6)	1645	0.3 (0.1-0.7)	0.0 (0.0-0.0)
	7.0 – 13.0	465	51.6 (45.8-57.4)	5.7 (3.5-7.8)	590	1.4 (0.1-2.6)	0.0 (0.0-0.0)
	13.1 – 25.0	301	54.6 (48.4-60.9)	3.9 (1.4-6.3)	342	2.1 (0.7-3.5)	0.2 (0.01-0.5)
	25.1 – 50.0	105	56.9 (46.3-67.5)	3.5 (0.2-7.2)	103	9.0 (1.2-16.9)	0.0 (0.0-0.0)
	>50.0	24	60.2 (34.8-85.6)	5.7 (0.5-16.9)	26	17.2 (0.5-34.0)	0.0 (0.0-0.0)

Data are % (95% CI) and were available on education, occupation and income for 4488, 4490 and 4467 subjects respectively.

Number of participants (N).

Hazardous drinking was very low in women. In men, the pattern of hazardous drinking was different to the pattern of current drinking among different levels of education. The group with the lowest level of education among males had the highest prevalence (8.5%) of hazardous drinking. Male ‘;senior managers and officials’ had the highest levels of hazardous drinking (11.1%) followed by the ‘;elementary occupations’ (7.3%). There were no participants with hazardous drinking among male ‘;professionals’ and only a few from ‘;armed forces’. Among the income categories; in males, higher prevalence of hazardous alcohol consumption was seen both in lower and higher income groups with the middle income groups having a lower prevalence.

### Smoking and alcohol use

Among the current smokers 70.1% were also drinking alcohol with 9.8% drinking at hazardous levels (Table 5). Among the ex-smokers, 41.8% had also given up drinking, 38.4% continued drinking with 4.7% at hazardous levels. Among those who had never smoked, 85.9% had been lifetime abstainers of alcohol, 9.8% were current drinkers and 0.3% were drinking at hazardous levels. Current smoking strongly and significantly correlated with both current drinking (Spearman r = 0.55, p < 0.0001) and hazardous drinking (Spearman r = 0.25, p < 0.0001).

**Table 5:** Cross-tabulation of drinking status and smoking in Sri Lankan adults

		**Drinking**				
		**Never**	**Former**	**Current - NH**	**Current - H**	p value*
**Smoking**	Never*	85.9%	4.3%	9.5%	0.3%	<0.0001
	Former*	19.8%	41.8%	33.7%	4.7%	<0.0001
	Current*	11.8%	18.1%	60.3%	9.8%	<0.0001

Never – lifetime abstainers, Former – past drinkers and smokers, Non-Hazardous (NH), Hazardous (H).

*p value for trend.

### Multiple logistic regression analysis in males

*Since the number of female drinkers was very low, results of multiple logistic regression analysis are presented with adjusted odds ratios (OR) only for males.* Current drinking was strongly associated with middle level of education (OR) 1.4 (95% CI 0.9-1.8) and high monthly income (OR) 2.2 (95% CI 0.8-5.9) (Table 6). Hazardous drinking was strongly associated with lower level of education (OR) 3.2 (95% CI 1.3-7.7). Current drinking (CD) and hazardous drinking (HD) together were positively associated with urban living (OR) 1.5(CD)/1.3(HD), Burgher ethnicity (OR) 2.3(CD)/7.8(HD) and current smoking (OR) 5.6(CD)/4.8(HD). Lifetime abstinence (Never drinker) was negatively associated with smoking (OR) 0.2 (95% CI 0.1-0.3), lower level of education (OR) 0.6 (95% CI 0.3-0.9) and urban living (OR) 0.7 (95% CI 0.5-0.9).

**Table 6:** Results of multiple logistic regression analysis for correlates of never, former, current and hazardous drinking in males

	**Total (n)**	**Never drinker**		**Former drinker**		**Current drinker**		**Hazardous drinker**	
		**OR**	**95% CI for OR**	**OR**	**95% CI for OR**	**OR**	**95% CI for OR**	**OR**	**95% CI for OR**
**Sector of residence**									
Rural	1448	1		1		1		1	
Urban	304	0.7	0.5-0.9	0.9	0.7-1.3	1.5	1.1-2.0	1.3	0.8-2.2
**Ethnicity**									
Sinhalese	1502	1		1		1		1	
Sri Lankan Tamil	58	1.5	0.8-2.8	0.3	0.1-0.9	1.3	0.7-2.4	0.3	0.07-1.4
Plantation Tamil	76	0.8	0.4-1.4	0.6	0.3-1.2	1.7	1.0-3.0	2.0	0.9-4.2
Muslim	113	12.7	1.7-21.0	0.9	0.5-1.4	0.04	0.02-0.09	0.0	0.0-0.0
Burgher	3	2.0	0.1-25.6	0.0	0.0-0.0	2.3	0.2-31.1	7.8	0.6-101.7
**Level of education**									
Less than 5 years	360	0.6	0.3-0.9	1.5	0.9-2.5	1.2	0.7-1.6	3.2	1.3-7.7
6 to 11 years	1085	0.6	0.4-0.8	1.2	0.8-1.8	1.4	0.9-1.8	2.4	1.1-5.2
≥ 12 years	307	1		1		1		1	
**Occupation**									
Senior officials and Managers	10	1.3	0.3-6.0	0.0	0.0-0.0	2.8	0.3-8.0	2.6	0.2-16.0
Professionals	49	1.0	0.5-1.9	0.9	0.4-2.0	1.2	0.6-2.3	0.0	0.0-0.0
Technical and Associated Professionals	75	0.6	0.3-1.2	0.4	0.1-0.9	2.6	1.4-4.7	1.4	0.5-4.2
Clerks	28	0.3	0.1-1.0	1.0	0.3-2.6	2.4	1.0-5.8	1.0	0.2-5.8
Sale and Service Workers	221	0.8	0.5-1.2	0.7	0.4-1.1	1.6	1.0-2.3	1.1	0.5-2.2
Skilled Agricultural and Fishery Workers	225	0.9	0.6-1.3	1.5	1.1-2.2	0.7	0.5-1.1	1.1	0.5-2.1
Craft and Related Workers	59	1.6	0.8-3.1	1.2	0.6-2.2	0.6	0.3-1.1	1.4	0.6-3.9
Plant and Mechanic Operators and Assemblers	100	0.7	0.4-1.3	0.6	0.3-1.2	1.8	1.0-3.1	1.1	0.4-2.6
Elementary Occupations	477	1.0	0.7-1.3	0.7	0.5-1.0	1.4	1.0-2.0	1.5	0.6-2.9
Armed Forces	11	1.4	0.3-5.9	1.0	0.2-4.8	0.8	0.2-3.1	0.0	0.0-0.0
No regular employment or unemployed	497	1		1		1		1	
**Age category**									
18-29 years	306	1		1		1		1	
30-39 years	329	0.4	0.3-0.6	0.9	0.6-1.5	2.1	1.5-3.1	2.2	0.9-.5.2
40-49 years	409	0.6	0.4-0.8	0.9	0.6-1.4	1.7	1.2-2.4	4.0	1.8-8.8
50-59 years	347	0.6	0.4-0.8	0.8	0.6-1.3	1.9	1.3-2.8	5.0	2.2-11.3
60-69 years	210	0.5	0.3-0.7	1.9	1.3-3.0	1.0	0.7-1.6	3.7	1.5-9.2
≥70 years	149	0.5	0.3-0.9	2.1	1.3-3.4	0.8	0.5-1.4	5.4	1.9-15.3
**Current Smoker**									
Yes	682	0.2	0.1-0.3	0.7	0.5-0.8	5.6	4.4-7.1	4.8	3.1-7.2
No	1070	1		1		1		1	
**Monthly income (LKR 000)**									
<7	861	1		1		1		1	
7.1-13.0	463	0.8	0.6-1.0	1.0	0.7-1.3	1.3	1.0-1.7	1.6	1.1-2.6
13.1-25.0	300	0.7	0.5-1.0	0.9	0.6-1.3	1.5	1.1-2.1	1.3	0.7-2.6
25.1-50.0	104	0.8	0.5-1.4	0.6	0.3-1.1	1.7	1.0-2.9	2.6	1.1-6.2
> 50.0	24	1	0.4-2.6	0.2	0.02-1.4	2.2	0.8-5.9	1.8	0.3-9.8

OR - Odds ratio, LKR – Sri Lankan Rupees.

## Discussion

The current alcohol use among males and females was 48.1% and 1.2% respectively. According to this study 65.7% were lifetime abstainers. A similar large study, conducted by the WHO for the World Health Survey (WHS -2003) in 2003 reported prevalence of 83.4%, 32.5% and 1.8% for overall abstinence, male current drinking and female current drinking respectively [10]. Therefore, our study carried out five years later shows an overall increase of alcohol use especially among males. However, the WHO GENACIS study conducted in 2002 – 2003 with a smaller sample size (n = 1201) showed a higher prevalence of current drinking both in men (53.1%) and women (6.4%) [9]. We recruited those who have abstained from drinking within the preceding six months as former drinkers. In other studies this was usually one year. This can underestimate the prevalence of current drinking in our study. The pattern of ethnic specific drinking in our study is similar to the data of the WHO GENACIS study.

According to the WHS -2003, the overall, male and female lifetime abstinence in India was 89.6%, 80.2% and 98.4% respectively [1]. Another large study in India among those above 10 years of age in 2005 reported male and female current drinking prevalences as 7.9% and 1.0% respectively. Compared with Sri Lanka, in Bangladesh which is a predominantly a Muslim country in South Asia, higher prevalence of lifetime abstinence were reported both for men (87.4%) and women (99.7%) (WHS -2003). In sharp contrast to the Sri Lankan data, the developed countries have much higher prevalence of alcohol use both in men and women; North America (Male 73%, Female 58%), Europe (Male 90%, Female 81%) and Western Pacific (Male 87%, Female 77%) [26]. The prevalence of alcohol use is lower than the Sri Lankan figures in predominantly Muslim countries in the Middle Eastern region (Iran and Saudi Arabia: Male 18%, Female 4%; Pakistan and Afghanistan: Male 17%, Female 1%) [26]. In a recent study from urban China, the prevalence of current drinking has been reported as 68% [27]. The above comparisons made with local and international data were done to identify a general trend and are interpreted with caution as the definitions used for drinking and populations studied, varied among these studies.

Our study showed that the middle aged males and young females have higher frequencies of drinking. The age specific alcohol consumption among males in our study is similar to the WHO GENACIS study conducted in Sri Lanka in 2002 and many other regional studies [9,28-30]. However, the higher prevalence of alcohol use among the young women in our study differs from the WHO GENACIS study and other regional studies [9,28,30] except for one study from Hong Kong [29].

According to our data, the majority of Sri Lankan adults; 79% of men and almost all women who consume alcohol drink less than 7 units per week on average. These data are similar to the previous findings from the WHO GENACIS study [9]. Among men 37.9% drink < 7, 2.6% between 7 – 14, 2.4% between 14 – 21 and 5.2% drink > 21 units of alcohol per week. The fact that there was no gradual decline in the frequency between the different categories of severity but a jump from the lowest to the highest would demonstrate the difficulty of maintaining drinking within non-hazardous/safe limits (male <21 and female <14) except for very light drinking due to the addictive properties of alcohol. Similar to our data, a low prevalence of hazardous drinking has been reported from a study from Hong Kong [29]. In contrast, much higher levels of hazardous drinking have been reported from the UK (31% of men and 20% of women consumed more than 21 units and 14 units of alcohol respectively) [31]. In the Australian workforce risky drinking (14–42 units per week) was seen in 7.4% of males and high risk drinking (>42 drinks per week) in 3.3% [32].

In the present study the highest prevalence of drinking in males was seen in those with medium level of education. In women, the highest current drinking was in those with the highest level of education. In contrast the hazardous drinking in males was highest in those with the lowest level of education. Our data for current drinking and education in males compares with the previous data from the WHO GENACIS study but was different in women in whom the highest prevalence of drinking was in those with middle level of education [9]. Similar data on the association of hazardous drinking and lower levels of education has been reported in many previous studies both from Asia and outside [28,29,32,33].

Among the different income groups, current drinking was higher in those with higher levels of income both in men and women. In contrast among males, hazardous drinking was equally high in both the higher and lower income groups with middle income groups having the lower prevalence. This has not been described in the previous studies on alcohol in Sri Lanka but similar pattern has been described both for current drinking and high risk drinking among the Australians [32]. In contrast to our data, higher prevalence of current drinking was reported in the low income groups in India [28]. Among the occupation groups both in men and women senior officials and managers had the highest prevalence of current drinking. Men in the same category had the highest prevalence of hazardous drinking followed by those engaged in elementary occupations and blue collar occupations. Hazardous drinking was very low among male professionals, clerks and the small number of participants from the armed forces. The higher prevalence of drinking in senior officials and managers and lower prevalence of hazardous drinking in those in the armed forces may partly be due to the bias created by the small number in these categories. In the study among the Australian work force higher prevalence of hazardous drinking was reported in the blue collar workers and white collar workers compared to professionals [32].

The current smoking prevalence is 38.0% among males and 0.1% among females older than 18 years in Sri Lanka [24]. Our study shows a strong association of smoking with both current drinking and hazardous drinking among Sri Lankan males. A significantly high proportion of quitters have given up drinking. Combined interventions for prevention of both smoking and alcohol use may have added benefits in this population. This has not been examined in the previously published studies from Sri Lanka but has been shown in studies from other countries [29,34,35].

The main strength of this study is the recruitment of a representative sample via cluster sampling with good response rate (91%). There are several limitations. Due to the prevailed war situation in the North and the East, the data on Sri Lankan Tamil and Moor ethnicities living on the other parts of the country may not represent the real picture on those ethnic groups. Comparisons and conclusions on certain ethnicities and occupations could have been affected due to the effect of small sample size. We classified those who have been drinking within the preceding six months as current drinkers. In other studies this was usually one year. This can underestimate the prevalence of current drinking compared to other studies. Alcohol is a social taboo especially among women in Sri Lanka. Therefore, there is a possibility of under reporting of current drinking among women although we used experienced and trained interviewers. Anecdotally there are small isolated pockets of high prevalence of female drinking in the North Western province in Sri Lanka. These pockets were not selected in the random selection process in our study. However since this is a very minor proportion of the overall Sri Lankan population, the effect on the overall results would be minimal. The alcohol content in illicit beverages is not standardized and, this may affect the accuracy of the calculation of units. Unavailability of drinking status of the 9% non-responders, recording only the total units of alcohol without the types of alcoholic drink and the drinking frequencies during data collection were among the other limitations.

The findings shown in this study have several implications in the management and prevention of alcohol use among adults in Sri Lanka. Nearly 50% of the males are current drinkers. This is unacceptably high in a predominantly Asian Buddhist culture which discourages use of alcohol according to the religion and cultural norms. The high prevalence of drinking in the higher social strata (higher education, income and occupational groups) both among men and women shows increasingly high acceptance of drinking even among women. These patterns can set unacceptable examples among wider community in Sri Lanka. Although highly educated people tend to avoid hazardous drinking, higher percentages of them continue to be current drinkers. In contrast higher percentages of poor and less educated indulged in hazardous drinking although the overall prevalence of alcohol consumption was lower than the highly educated. We feel that this can trigger a vicious cycle of increasing poverty, violence, fragmentation of families and other harmful consequences of alcohol abuse in the poor and less educated segments in the society compared to the educated. All highly educated were not rich, as income levels do not always increase with the level of education in Sri Lanka (professionals work in the government sector). This explains why hazardous and current drinking was evenly spread over all income categories. Multiple factors may have contributed to higher current drinking in the urban educated groups. They have a higher disposable income, more opportunities for consumption in terms of social gatherings, and also use alcohol as a means of socializing during business meetings.

The associations between smoking and drinking indicate that those who continue one habit continues the other and those who give up tend to give up both. This phenomenon needs to be taken into consideration in future preventive programmes on smoking and drinking cessation and prevention.

## Conclusions

In summary, this study has presented new data on alcohol consumption epidemiology for Sri Lankan adults with insights to preventive action. There has been a change in the patterns of alcohol use in women with the prevalence being higher in the younger, more educated and affluent women compared to the previous studies. Nearly half of the adult males consume alcohol in Sri Lanka. Future action on prevention and control of alcohol use needs to focus on these new data and the risk factors for drinking in the Sri Lankan population. Effective interventions to prevent the rising prevalence of drinking in males and in educated high income women, will most likely help avert an epidemic of drinking in Sri Lanka.

## Abbreviations

WHO: World Health Organisation; GENACIS: Gender, Alcohol and Culture: An International Study; SLDCS: Sri Lanka Diabetes and Cardiovascular Study; LKR: Sri Lankan Rupees; ISCO: International Standard Classification of Occupation; USA: Untied States of America; UK: United Kingdom.

## Competing interests

The authors declare that they have no competing interests.

## Authors’ contributions

PK participated in the design of the study, data collection, examining the participants, performed the statistical analysis and drafted the manuscript. NK, AR did data collection, examining the participants and helped to draft the manuscript. CR, RS, DM participated in its design and helped to draft the manuscript. All authors read and approved the final manuscript.

## Pre-publication history

The pre-publication history for this paper can be accessed here:

http://www.biomedcentral.com/1471-2458/14/612/prepub

## Supplementary Material

Additional file 1 **Sri Lanka Diabetes and Cardiovascular diseases Study (SLDCS) -Questionnaire.** Interviewer administered questionnaire used to collect data. Click here for file

Additional file 2 **Quantification of Units of alcohol consumed within a week.** Annexure to the main questionnaire. Each interviewer carried this annexure to help calculate the units of alcohol consumed. Click here for file

Additional file 3 Calculation of the number of units of different types of alcohol in Sri Lanka- Reference table carried by each interviewer. Click here for file
