# Supplementary material for: Prevalence, patterns and correlates of alcohol consumption and its’ association with tobacco smoking among Sri Lankan adults: a cross-sectional study
Source: BMC Public Health. 2014 Jun 17;14:612. doi: 10.1186/1471-2458-14-612 (PMC4074142; doi:10.1186/1471-2458-14-612)
Supplement: Additional file 1 — Sri Lanka Diabetes and Cardiovascular diseases Study (SLDCS) -Questionnaire. Interviewer administered questionnaire used to collect data. [file 1471-2458-14-612-S1.pdf]

Form No: A Study ID: | E | | | | | | | | |

Page 1 of 1 Initials: | | | |

<u>Confidential: Information on Form A should not to be entered into the main database</u>

SRI LANKA DIABETES AND CARDIOVASCULAR DISEASES STUDY .
(SLDCS)
Questionnaire

Contact Details (Please print in block capitals)

Title: Mr/Miss/Mrs/Dr/Rev (delete not applicable)

Surname with initials: ……………………………………………………………………
(Eg: Ranasinghe L.M.S)

Other Names: ………………………………………………………………………………

………………………………………………………………………………

Initials (upto three): | | | | (Eg. L. M. S.)
(when there are more than three initials always take first three)
(When there are less than three take the first letter of the surname as the last initial)

Permanent Address:
No ………….
Street ……………….………………………..…………………………………………….
City …………………..……………………………..……………………………………

Tel No (Res): ……………………………..
Tel No (Off): ……………………………..
Mobile No: ……………………………..
Email: ……………………………..

Corresponding address (if different from above)

No ………….
Street ……………………………………..…………………………………………
City ……………………………………..…………………………………………

Tel No (Res): ……………………………..
Tel No (Off): ……………………………..
Mobile No: ……………………………..
Email: ……………………………..

Measure blood pressure - 1st reading

HISTORY

**Date of interview** :………………………..	dd / mm / yyyy	(1)
**District** :…………………………………		
**District code:** (refer to appendix 1 to get the district code)		(2)
**Cluster name** :……………………………………		
**Cluster number:** (refer to appendix 2 to get the cluster number)	**Cluster No**	(3)

Sector

: Rural	1	1	
Urban	2	2	(4)
Estates	3	3	

Sector (Subjective)

: Rural	1	1	
Urban	2	2	(5)
Estates	3	3	

Date of birth : dd / mm / yyyy (6)

Age : (calculate later) months / years (7)

Circle the correct response

Sex :

Male	1	1	(8)
Female	2	2	

Ethnicity :

1	Sinhalese	1	
2	Sri lankan Tamil	2	
3	Indian Tamil	3	(9)
4	Muslim	4	
5	Burger	5	
6	Other	6	

Marital Status :

1	Unmarried	1	
2	Married	2	
3	Divorced	3	(10)
4	Widowed	4	
5	Seperated	5	

Fasting Status

Time at your last meal or drink (except water): (24 hour clock) — Hour / e (11)

Time at which your blood sample was taken: (24 hour clock) — Hour / e (12)

Socio-Economic Factors

Highest educational level achieved:

Circle the correct response

1	No formal education	1	
2	Grade 1-5	2	
3	Grade 6-11	3	(13)
4	Qualified GCE (O/L)	4	
5	Qualified GCE (A/L)	5	
6	Graduate/Diploma	6	
7	Postgraduate	7	
8	Other	8	

HISTORY

	Circle the correct response	
**Your present occupation:(If in doubt refer to annexure 2)**		
1 Senior Officials and Managers	1	
2 Professionals	2	
3 Technical and Associate Professionals	3	
4 Clerks	4	
5 Sales and Service workers	5	(14)
6 Skilled agricultural and Fishary worker	6	
7 Craft and Related trades workers	7	
8 Plant and machine operators and assemblers	8	
9 Elementary occupations	9	
10 Armed Forces	10	
11 Unemployed	11	
12 Other (specify)............................	12	
**Duration of present occupation:**	years [ ][ ] months [ ][ ]	(15)
**Sector of the place of employment** 1 Rural	1	(16)
2 Urban	2	
**Monthly family income**	**Circle the correct response**	
1 Less than Rs 6999	1	
2 Rs 7000- 12999	2	
3 Rs 13000- 24999	3	(17)
4 Rs 25000- 49999	4	
5 More than Rs 50000	5	
**Foreign stay** 1 Yes	1	(18)
2 No	2	
**Duration (Months)**	[ ][ ][ ]	(19)
**Smoking**	**Circle the correct response**	
1 Never	1	
2 Ex-smoker (not for last >6 months)	2	
3 1 or <1 per day	3	(20)
4 Currently <10 per a day	4	
5 Currently 10 - 20 per a day	5	
6 Currently >20 per a day	6	
**Alcohol- quantity (Refer to annexure 3)**		
How much (units of alcohol) do you drink per week on average?	**Circle the correct response**	
1 Nill	1	
2 Ex-drinker (not for last >6 months)	2	
3 < or = 3 per week	3	(21)
4 4-7 per day	4	
5 7-14 per week	5	
6 15-21 per week	6	
7 22 -35 per week	7	
8 >36 per week	8	

HISTORY

Physical Activity Level - IPAQ score (Refer to annexure 4)

Do you engage in vigorous physical activities ? — **Circle the correct response**

(for occupation, exercise/sports, leisure) -- please underlying the activity — **yes** 1 **no** 0 (22)

eg: carrying heavy weights, fast cycling, swimming, digging the earth, heavy construction work, running, aerobics, chopping wood, heavy manual labour, exercise in a gym, etc

If yes,

Number of days of vigorous activities per week? ☐ (23)

Average minutes per day of vigorous activities? ☐☐☐ min (24)

Met/Min per week by vigorous physical activities? ☐☐☐☐ Met/Min (25)

(Met/Min = 8 * mins of vigorous activities * days per week)

Do you engage in moderate physical activities ? — **Circle the correct response**

(for occupation, exercise/sports, leisure) -- please underlying the activity — **yes** 1 **no** 0 (26)

eg: carrying light weights, slow cycling, washing, sweeping the garden and house, scrubbing the floor

If yes,

Number of days of moderate activities per week? ☐ (27)

Average minutes per day of moderate activities? ☐☐☐ min (28)

Met/Min per week by moderate physical activities? ☐☐☐☐ Met/Min (29)

(Met/Min = 4 * mins of moderate activities * days per week)

Circle the correct response

Do you walk more than 10 mins per day at a stretch ? — **yes** 1 **no** 0 (30)

(for occupation, exercise/sports, leisure)

If yes,

Number of days of walking per week? ☐ (31)

Average minutes per day of walking? ☐☐☐ min (32)

Met/Min per week by walking? ☐☐☐☐ Met/Min (33)

(Met/Min = 3.3 * mins of walking * days per week)

Physical activity level: (calculate later) — **Circle the correct response** (34)

1	Insufficiently active (sedentary)	1
2	Moderately active	2
3	HEPA active	3

Do you have following symptoms?

	Yes	No	
Polyuria…………………………………………..	1	0	(35)
Nocturia…………………………………………..	1	0	(36)
Thirst……………………………………………	1	0	(37)
Weight loss……………………………………..	1	0	(38)
Wound infection……………………………….	1	0	(39)
Cellulitis and abscess…………………………….	1	0	(40)
Balanitis…………………………………………	1	0	(41)
Giddiness………………………………………….	1	0	(42)
Sleepiness………………………………………..	1	0	(43)
Blurring of vision………………………………..	1	0	(44)

HISTORY

Ask only from females

	Yes	No	
**History of gestational diabetes:**	1	0	(45)
**Have you delivered a baby with birth weight > 4 kg (9lbs)?**	1	0	(46)

Ask only from females - History of..

	Yes	N/A	No	
subfertility	1	2	0	(47)
amenorrhoea/oligomenorrhoea	1	2	0	(48)
Diagnosis of polycystic ovarian disease	1	2	0	(49)

	Yes	No	
**Steroids therapy during the last year**	1	0	(50)

If yes,

mode of therapy — Circle the correct response

Systemic	1	
inhaled	2	(51)
Skin	3	
Other topical forms	4	

duration Months/years mm | yy [ ][ ] [ ][ ] (52)

Have you ever been told by a doctor that you are suffering from any of the conditions stated below?

(clarify with the diagnosis card if available)

Definite-D May present - M Absent-A

	D	M	A	
Hypertension……..……………………………………..	1	2	0	(53)
Ischaemic Heart Diseases…..………………………	1	2	0	(54)
Cerebrovascular Disease……………………………	1	2	0	(55)
Peripheral vascular disease………………………..	1	2	0	(56)
Hyperlipidaemia…………………………………..	1	2	0	(57)
Obesity……………………….…….………….	1	2	0	(58)
Pancreatic disease………………………………….	1	2	0	(59)
Autoimmune diseases : (adissons, hypothyroidism, hypoparathyroidism, alopecia areata, vitiligo)	1	2	0	(60)
Other (specify) 1 ………………………				(61)
2 ………………………				(62)

Cardiovascular Symptoms

	Yes	No	
* Have you ever had any pain or discomfort or any pressure or heaviness in your chest?	1	0	(63)
* Do you get the pain in the centre of the chest or left arm?	1	0	(64)
* Do you get it when you walk at an ordinary pace on level or when walk uphill or hurry?	1	0	(65)
* Do you slow down if you get the pain while walking?	1	0	(66)
* Does the pain go away if you stand still or if you take a tablet under the tongue?	1	0	(67)
* Does the pain go away in less than 10 minutes?	1	0	(68)
* Have you ever had a severe chest pain across the front of your chest lasting for half an hour or more ?	1	0	(69)

HISTORY

	Yes	No		
**Peripheral vascular disease**	**Yes**	**No**		
* Pain in the calf during walking which is relieved by stopping?	1	0		(70)
**Stroke/TIA**	**Yes**	**No**		
* Have you ever had any of the following: difficulty in talking, weakness of arm and/ or leg on one side of the body or numbness on one side of the body?	1	0		(71)
**Neurological Symptoms (Diabetic Neuropathy Symptom Score)**	**Yes**	**No**		
* Are you suffering from unsteadiness in walking? (need for visual control, increase in the dark, walk like a drunk man, lack of contact with floor)	1	0		(72)
* Do you have a burning, aching pain or tenderness at your legs or feet? (occuring at rest or at night, not rtelated to exercise, exclude intermittent claudication)	1	0		(73)
* Do you have prickling sensations at your feet or legs? (occuring at rest or at night, distal>proximal, stocking distribution)	1	0		(74)
* Do you have places of numbness in your feet or legs?	1	0		(75)
* Have you had non-healing ulcers?	1	0		(76)
* Are you sexually active?	1	0		(77)
* Do you have any sexual problems?	1	0		(78)
If yes, check the correct answer	**Yes**	**No**		
Lack of desire	1	0		(79)
Impotence(males only)/ reduced lubrication (females)	1	0		(80)
Lack or delay of ejaculation/ lack or delay of orgasm in females	1	0		(81)
Pain during intercourse	1	0		(82)
Other:……………………	1	0		(83)

Family history of diabetes:

	Yes	No	Don't know	
Has anyone in your immediate family ever had " Diabetes"?	1	0		(84)
Indicate the diabetic status of the following relatives :	**Yes**	**No**	**Don't know**	
Father	1	0	2	(85)
Mother	1	0	2	(86)
Maternal grand mother	1	0	2	(87)
Maternal grand father	1	0	2	(88)
Paternal grand mother	1	0	2	(89)
Paternal grand father	1	0	2	(90)

Age of onset of diabetes in father		(91)
Age of onset of diabetes in mother		(92)
Total number of male siblings		(93)
Number of male siblings with diabetes		(94)
Average age of onset of brothers		(95)
Total no of female siblings		(96)
Number of female siblings with diabetes		(97)
Average age of onset of sisters		(98)
Total number of male children		(99)
Number of male children with diabetes		(100)
Average age of onset in sons		(101)
Total no of female children		(102)
Number of female children with diabetes		(103)
Average age of onset in daughters		(104)

HISTORY

	Yes	No	Don't know	
**Family history of hypertension:**				
Has anyone in your immediate family ever had " hypertension"?	1	0		(105)
Indicate the hypertension status of the following relatives :	**Yes**	**No**	**Don't know**	
Parents	1	0	2	(106)
Siblings	1	0	2	(107)
Grand parents	1	0	2	(108)
Children	1	0	2	(109)
**Family history of ischaemic heart diseases:**	**Yes**	**No**		
Has anyone in your family ever had " ischaemic heart diseases"?	1	0		(110)
Indicate the history of IHD of the following relatives :	**Yes**	**No**	**Don't know**	
Parents	1	0	2	(111)
Siblings	1	0	2	(112)
Grand parents	1	0	2	(113)
Children	1	0	2	(114)
**Family history of strokes:**	**Yes**	**No**		
Has anyone in your family ever had " strokes"?	1	0		(115)
Indicate the history of stroke of the following relatives :	**Yes**	**No**	**Don't know**	
Parents	1	0	2	(116)
Siblings	1	0	2	(117)
Grand parents	1	0	2	(118)
Children	1	0	2	(119)

Measure blood pressure - 2nd reading

Form No:	B	2	Study ID:	E
Page	1 of 2		Initials:	

EXAMINATION & INVESTIGATIONS

Height (cm) :……………………………… (120)

Weight (Kg) :……………………………… • (121)

BMI (calculate later) :…………………………….. • (122)

Waist circumference – cm :…………………………… (123)

Hip circumference – cm :……………………………. (124)

Waist - Hip ratio :…….……………………. • (125)

Distance from xyphisternum to umbilicus (cm) : ………………… (126)

Blood Pressure (mmHg)

	Systole		Diastole	
First reading		/		(127)
Second reading (10 min later)		/		(128)

	Yes	No	
Acanthosis nigricans :	1	0	(129)

Foot ulcers

Circle the correct response

Active	1	
Healed	2	(130)
None	0	

	Yes	No	
Lipoatrophy:	1	0	(131)

Features of

	Yes	No	
* Hirsutism (females only)	1	0	(132)
* Dysmorphism	1	0	(133)
* Acromegaly	1	0	(134)
* Cushings syndrome	1	0	(135)
* Hyperthyroidism	1	0	(136)

***Any patient with these features needs to be shown to the PI**

To be filled when laboratory tests are available

Fasting blood glucose level at this visit - mg/dl (137)

2 hour post glucose blood glucose at this visit- mg/dl (138)

Lipid profile - mmol/l

Total cholesterol • (139)

HDL cholesterol • (140)

LDL cholesterol • (141)

Triglycerides • (142)

FORM NO 2 : EXAMINATION & INVESTIGATIONS

ALT (iu/L)	☐☐☐	(143)
AST (iu/L)	☐☐☐	(144)

ECG
- Normal 1
- Ischaemic changes present 2 (145)
- Specify ischaemic changes 3

LVH present(voltage criteria) …………………………..

Yes	No	
1	0	(146)

Data completed:
1. History ☐
2. Anthropometry ☐
3. Fasting blood samples ☐
4. Post glucose load blood sample ☐
5. ECG ☐

…………………..
Signature:

To be completed by the Principal Investigator Before data entry

Completion of the questionnaire satisfactory

Yes 1 ☐
No 2 ☐

………………………………………..
Signature of the Principal Invesigator
