# Supplementary material for: Prevalence, patterns and correlates of alcohol consumption and its’ association with tobacco smoking among Sri Lankan adults: a cross-sectional study
Source: BMC Public Health. 2014 Jun 17;14:612. doi: 10.1186/1471-2458-14-612 (PMC4074142; doi:10.1186/1471-2458-14-612)
Supplement: Additional file 2 — Quantification of Units of alcohol consumed within a week. Annexure to the main questionnaire. Each interviewer carried this annexure to help calculate the units of alcohol consumed. [file 1471-2458-14-612-S2.pdf]

Additional file 2

Study ID | E | | | | | | | | |

Sri Lanka Diabetes and Cardiovascular Diseases Study (SLDCS)

(Annexure 3) to Main Questionnaire

Quantification of Units of alcohol consumed within a week

- Have you taken an alcoholic drink during the last 6 months? Yes ☐

  No ☐

- If the answer to the above question is **Yes,** indicate the **type** of alcoholic drink you **mostly** consume and the quantity **per week**

**Type of alcoholic beverage**	**Quantity per single consumption** (Drinks, Glasses, Pints)	**Frequency per week** (How many times do you drink per week?)	**Total units* consumed per week by each beverage**
Arrack	…………..... (drinks)<br>*(1 drink= 25-30 ml)*	....	……………….
Whisky	..………….... (drinks)<br>*(1 drink= 25-30 ml)*	....	……………….
Other**	.....………… (drinks)<br>*(1 drink= 25-30 ml)*	....	……………….
Illicit spirits	..………….. .. (drinks)<br>*(1 drink= 50 ml)*	....	……………….
Wine	……. … (glasses of wine)<br>*(1 glass of wine= 175 ml)*	....	……………….
Beer	…………….. (Pints)<br>*(1 pint= 200-250 ml)*	....	……………….
Toddy	…………….. (Pints)<br>*(1 pint= 200-250 ml)*	....	……………….

Total units consumed per week : **……………….**

*1 unit of alcohol= 1 drink of arrack/whisky, 2 drinks of illicit spirit,1 small glass of wine, half a pint of beer/toddy (ref- Calculation of the number of units of different types of alcohol in Sri Lanka-Reference table)

** Other alcoholic spirits/beverages such as brandy, gin, vodka.
