# Supplementary material for: Prevalence, patterns and correlates of alcohol consumption and its’ association with tobacco smoking among Sri Lankan adults: a cross-sectional study
Source: BMC Public Health. 2014 Jun 17;14:612. doi: 10.1186/1471-2458-14-612 (PMC4074142; doi:10.1186/1471-2458-14-612)
Supplement: Additional file 3 — Calculation of the number of units of different types of alcohol in Sri Lanka- Reference table carried by each interviewer. [file 1471-2458-14-612-S3.pdf]

Additional file 3

Sri Lanka Diabetes and Cardiovascular Diseases Study (SLDCS)

Calculation of the number of units of different types of alcohol in Sri Lanka

(Reference Table)

Type of Alcohol	Pure alcohol % by volume	A single unit in ml	1 unit in conventional measurements
Arrack	34-36	30	One drink
Illicit spirits*	20(approx.)	50	Two shots/drinks
Beer	4.5-5	200-250	Half a pint
Toddy	5	200-250	Half a pint
Whisky	40-43	25	One drink
Wine	11-12	175	One small glass

*Includes ‘Kassippu’ (Moon shine/homemade alcohol)
